# Teachers of Anatomy and Surgery

**Published:** 1836-10

**Authors:** Edmund Balfour

**Affiliations:** Sec.


### TEACHERS OF ANATOMY AND SURGERY.

*To Gentlemen applying for the recognition of the Royal College of Surgeons in London, as Teachers of Anatomy and Surgery.*

The Council of the Royal College of Surgeons deem it essential that teachers of anatomy should be provided with the preparations of parts mentioned in the following schedule, intended to comprise those preserved, injected, or otherwise prepared structures, which cannot be adequately demonstrated in the recent subject.

1. Artificial skeletons, male and female.
2. The several bones of the skeleton, including the separated bones of the cranium.
3. Sections of the cranium.
4. Preparations showing the structure and growth of bone.
5. The component structures of the various joints.
6. The deciduous and permanent teeth, their structure and formation.
7. The mouth, salivary glands, fauces, and other parts concerned in deglutition.
8. The organs of digestion and their appendages; exhibiting the structure of the alimentary canal, of the glandular and other parts concerned in the digestive process.
9. The thoracic duct, the lacteals, and the other absorbent vessels, with their glands.
10. The heart, exhibiting its structure in the adult and fœtal states; and the parts concerned in the circulation of the fœtus.
11. The blood-vessels, their structure, arrangement, and distribution.
12. The larynx, the trachea, and the lungs, with the distribution of the air-tubes and the blood-vessels.
13. The brain and spinal chord, with their membranes.
14. The nerves, their origin, structure, and distribution.
15. The organs of the senses.

1. Of sight: the globe of the eye, its component textures, the lachrymal apparatus, and its other appendages.
2. Of hearing: the various parts comprised in its external, middle, and internal divisions.
3. Of taste: the tongue, its nerves and papillæ.
4. Of smell: the nasal chambers, the communicating sinuses and their lining membranes.
5. Of touch: the peculiar conformation of the skin instrumental thereto, with the structure of the common integuments and their appendages.

16. The urinary organs, showing the structure of the kidney and ureters, of the bladder, the urethra, and Cowper's glands.
17. The male organs of generation, exhibiting the structure of the testis and vas deferens, of the vesiculæ seminales, of the prostate gland, and of the penis.
18. The female organs of generation in the unimpregnated and gravid state.
19. The peculiarities of the fœtus.

It is expected that teachers of surgery should have the means of illustrating by suitable preparations those points in pathology and practice which are treated of in surgical lectures.

March 15th, 1836. EDMUND BALFOUR, Sec.

---

